# Poly[octakis(1*H*-imidazole-κ*N* ^3^)octa-μ-oxido-tetra­oxidodicopper(II)tetra­vanadate(V)]

**DOI:** 10.1107/S1600536812008252

**Published:** 2012-03-03

**Authors:** Songwuit Chanthee, Tongchai Saesong, Watcharin Saphu, Kittipong Chainok, Samroeng Krachodnok

**Affiliations:** a Department of Chemistry, Faculty of Science, Naresuan University, Muang, Phitsanulok 65000, Thailand; b Department of Applied Chemistry and Center for Innovation in Chemistry, Faculty of Science, Lampang Rajabhat University, Lampang 52100, Thailand

## Abstract

In the title inorganic–organic hybrid compound, [Cu_2_V_4_O_12_(C_3_H_4_N_2_)_8_]_*n*_, the V^V^ ion is tetra­coordinated by four O atoms and the Cu^II^ ion is hexa­coordinated by four N atoms from four imidazole ligands and two O atoms from two tetra­hedral vanadate (VO_4_) units in a distorted octa­hedral geometry. The structure consists of two-dimensional sheets constructed from centrosymmetric cyclic [V_4_O_12_]^4−^ anions covalently bound through O to [Cu(imidazole)_4_]^2+^ cations. Adjacent sheets are linked by N—H⋯O hydrogen bonds and weak C—H⋯π inter­actions (H⋯centroid distances = 2.59, 2.66, 2.76, 2.91 and 2.98 Å into a three-dimensional supra­molecular network.

## Related literature

For background to inorganic–organic hybrids involving vanadium oxides, see: Cheetham *et al.* (1999 ▶); Hagrman *et al.* (2001 ▶); Natarajan & Mandal (2008 ▶); Zavalij & Whittingham (1999 ▶). For related structures, see: Chainok *et al.* (2011 ▶). For the bond valence sum calculation, see: Brown & Altermatt (1985 ▶).
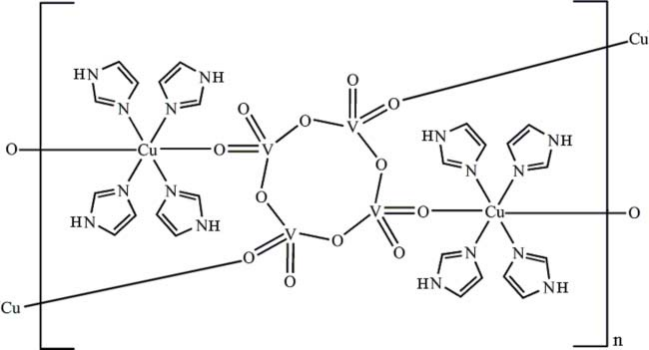


## Experimental

### 

#### Crystal data

[Cu_2_V_4_O_12_(C_3_H_4_N_2_)_8_]
*M* *_r_* = 1067.52
Monoclinic, 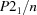

*a* = 10.1761 (6) Å
*b* = 16.5092 (9) Å
*c* = 12.0372 (7) Å
β = 103.844 (1)°
*V* = 1963.50 (19) Å^3^
*Z* = 2
Mo *K*α radiation
μ = 2.05 mm^−1^
*T* = 100 K
0.24 × 0.20 × 0.18 mm

#### Data collection

Bruker APEX CCD diffractometer
Absorption correction: multi-scan (*SADABS*; Sheldrick, 1996 ▶) *T*_min_ = 0.639, *T*_max_ = 0.722
11719 measured reflections
4370 independent reflections
3830 reflections with *I* > 2σ(*I*)
*R*_int_ = 0.025

#### Refinement

*R*[*F* ^2^ > 2σ(*F* ^2^)] = 0.028
*wR*(*F* ^2^) = 0.073
*S* = 1.04
4370 reflections
262 parameters
H-atom parameters constrained
Δρ_max_ = 0.51 e Å^−3^
Δρ_min_ = −0.30 e Å^−3^

### 

Data collection: *SMART* (Bruker, 2007 ▶); cell refinement: *SAINT* (Bruker, 2007 ▶); data reduction: *SAINT*; program(s) used to solve structure: *SHELXS97* (Sheldrick, 2008 ▶); program(s) used to refine structure: *SHELXL97* (Sheldrick, 2008 ▶); molecular graphics: *ORTEP-3* (Farrugia, 1997 ▶) and *DIAMOND* (Brandenburg, 1999 ▶); software used to prepare material for publication: *publCIF* (Westrip, 2010 ▶).

## Supplementary Material

Crystal structure: contains datablock(s) global, I. DOI: 10.1107/S1600536812008252/hy2518sup1.cif

Structure factors: contains datablock(s) I. DOI: 10.1107/S1600536812008252/hy2518Isup2.hkl

Supplementary material file. DOI: 10.1107/S1600536812008252/hy2518Isup3.cdx

Additional supplementary materials:  crystallographic information; 3D view; checkCIF report

## Figures and Tables

**Table 1:** Hydrogen-bond geometry (Å, °) *Cg*1, *Cg*2, *Cg*3 and *Cg*4 are the centroids of the C11/C12/N13/C14/C15, C21/C22/N23/C24/C25, C31/C32/N33/C34/C35 and C41/C42/N43/C44/C45 rings, respectively.

*D*—H⋯*A*	*D*—H	H⋯*A*	*D*⋯*A*	*D*—H⋯*A*
N13—H13⋯O3^i^	0.88	2.01	2.827 (2)	155
N23—H23⋯O5^ii^	0.88	1.95	2.779 (2)	155
N33—H33⋯O2^iii^	0.88	1.90	2.694 (2)	149
N43—H43⋯O5^iv^	0.88	1.88	2.701 (2)	155
C24—H24⋯*Cg*1^v^	0.95	2.99	3.912 (2)	165
C12—H12⋯*Cg*2^i^	0.95	2.59	3.429 (2)	147
C22—H22⋯*Cg*3^vi^	0.95	2.66	3.332 (2)	128
C44—H44⋯*Cg*3^vii^	0.95	2.91	3.816 (2)	161
C32—H32⋯*Cg*4^viii^	0.95	2.76	3.321 (2)	119

Symmetry codes: (i) 
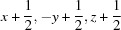
; (ii) 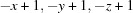; (iii) 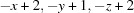; (iv) 
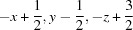
; (v) 
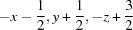
; (vi) 
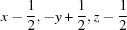
; (vii) 
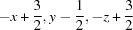
; (viii) 
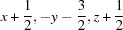
.

**Fig. 1.**
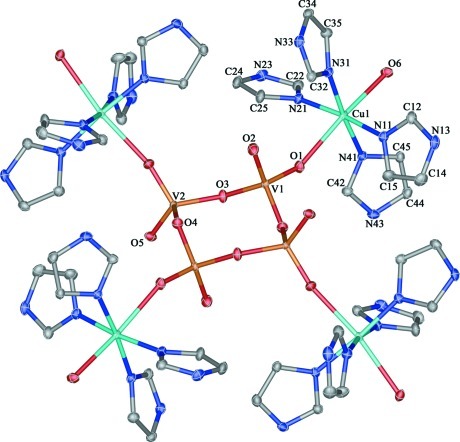
The aymmetric unit of (I). Displacement ellipsoids are drawn at the 50% probability level. H atoms have been omitted for clarity.

**Fig. 2.**
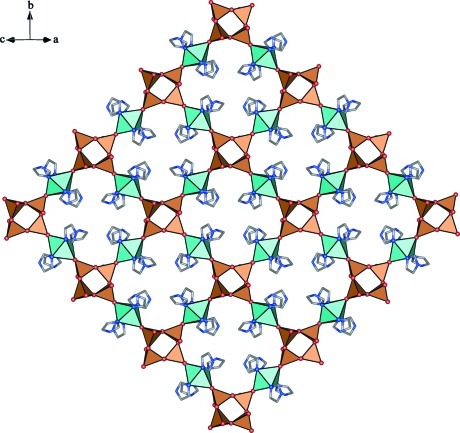
Polyhedral representation of the two-dimensional sheet structure of (I). H atoms have been omitted for clarity.

**Fig. 3.**
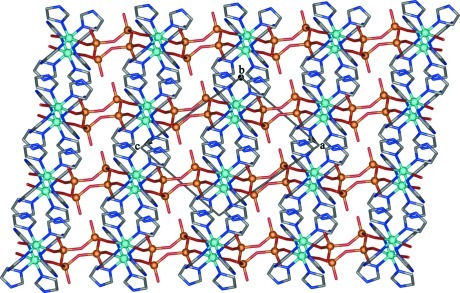
View of the layer structure of (I) along the b axis. H atoms have been omitted for clarity.

**Fig. 4.**
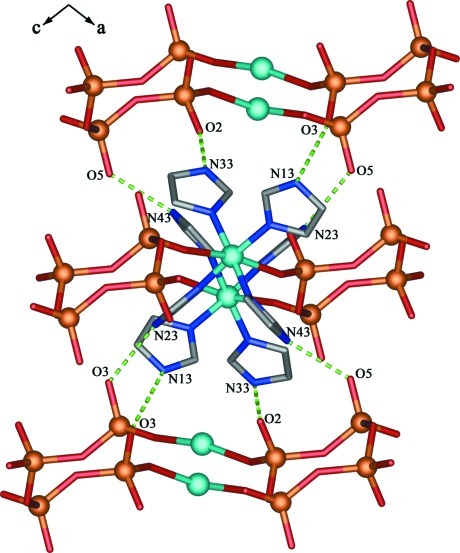
View of N—H···O hydrogen bonds (dashed lines) in (I). H atoms have been omitted for clarity.

**Fig. 5.**
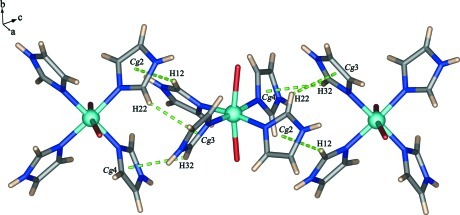
View of weak intermolecular C—H···π interactions (dashed lines) in (I). (H12···Cg2 = 2.59, H22···Cg3 = 2.66, H44···Cg3 = 2.91, H32···Cg4 = 2.76 Å.)

## supplementary crystallographic information

### Comment

The synthesis of extended solids based on inorganic-organic hybrids involving vanadium oxides have received much attention due to their structural diversity and fascinating properties with applications in catalysis, ion exchange, magnetic materials (V^4+^, V^3+^), and the development of new cathode materials for Li batteries (Cheetham *et al.*, 1999; Natarajan & Mandal, 2008). One approach for the design of such hybrid vanadium oxide frameworks is to introduce organic amine ligands to the secondary metal sites incorporating hydrothermal or solvothermal methods. Following this approach, numerous hybrid metal vanadium oxide framework structures involving edge- and corner-sharing VO_4_ tetrahedra, VO_5_ square-pyramids, or VO_6_ octahedra and incorporating organic species such as diammonium cations have been successfully synthesized (Hagrman *et al.*, 2001). Among the solid state polyhedra of vanadium oxide structures, the tetrahedral linking unit VO_4_ is commonly observed (Zavalij & Whittingham, 1999). Recently, we have used the aromatic diamine planar geometry of imidazole and hydrothermal method to synthesize a new series of hybrid inorganic-organic metal vanadium oxide compounds with a general formula [*M*(imidazole)_4_V_2_O_6_]_n_ (*M* = Mn, Co, Ni) (Chainok *et al.*, 2011). The structures of these isostructural species contain two-dimensional polymeric sheets and display an interesting order-disorder crystallographic phase transition between the *P*4_2_/*n* at 295 K and *I*4_1_/*a* (Mn and Co) or *P*2/*n* (Ni) space groups at 100 K. As a continuation of this work, we report the title compound, (I), a new member of this family, which is isostructural to [*M*(imidazole)_4_V_2_O_6_]_n_.

Fig. 1 shows the asymmetric unit with atomic numbering and the detailed coordination environment for the metals ions in (I). It crystallizes in the monoclinic space group *P*2_1_/*n* at 100 K. There are two crystallographically independent V atoms and one distinct Cu atom. The bond valence sum calculation (Brown & Altermatt, 1985) indicates that the oxidation state of all the V atoms are pentavalent and Cu is divalent. All the V atoms adopt a tetrahedral geometry involving one terminal and three bridging O atoms. The mean V—O bond lengths involving the O atoms bridging V and Cu atoms are 1.632 Å and significantly shorter than those involving the O atoms bridging V atoms only (mean 1.818 Å). The mean V—O bond lengths of those involving terminal O atoms are 1.648 Å. The bond lengths and angles around the V atoms are comparable to those observed in the ordered phases of [*M*(imidazole)_4_V_2_O_6_]_n_ (Chainok *et al.*, 2011). The Cu atom is in a distorted octahedral geometry with four equatorial N atoms from the imidazole ligands and two axial O atoms from VO_4_ units. The mean M—N bond lengths in the [*M*(imidazole)_4_V_2_O_6_]_n_ series are 2.251, 2.137, 2.137 and 2.008 Å for the Mn, Co, Ni and Cu complexes, respectively, showing that the M—N bond lengths systematically decrease with increasing atomic number in the order of Mn^II^ > Co^II^~ Ni^II^ > Cu^II^. A similar trend, however, is not observed for the M—O bond lengths. The mean M—O bond lengths for the Mn, Co and Ni complexes are 2.154, 2.100 and 2.100 Å, respectively, while the Cu complex has the longest M—O bond lengths with a mean value of 2.413 Å. The latter case seems to be influenced by the distortion of Jahn-Teller effect on the Cu^II^ coordination polyhedra.

As shown in Fig. 2, the crystal structure consists of two-dimensional polymeric sheets constructed by centrosymmetric cyclic V_4_O_12_ tetramers (exhibiting an approximate *C*_2v_ symmetry) and CuN_4_O_2_ octahedra involving four separate imidazoles. The framework of sheet structure can be alternatively described as four corner-connected VO_4_ tetrahedra forming eight-membered [V_4_O_4_] small rings, and four CuN_4_O_2_ octahedra and eight VO_4_ tetrahedra further connected to form twenty four-membered [Cu_4_V_8_O_12_] large rings. A packing view along the *b* axis (Fig. 3) shows the two-dimensional layer structure with the imidazole ligands decorating the layer on the periphery. The coordinated imidazole ligands on the Cu atoms form intermolecular N—H···O hydrogen bonds to the bridging oxido groups and the terminal vanadyl sites above and below adjacent layers (Fig. 4, Table 1). These layers are further stabilized by weak edge to face C—H···π interactions between the imidazole H atoms (H12, H24, H32, and H44) and the centroids (*Cg*) of the imidazole rings (Fig. 5). It should be noted that there is no π–π stacking between adjacent imidazole rings in this compound.

### Experimental

A mixture of Cu(CH_3_COO)_2_.2H_2_O (180 mg, 1.0 mmol), V_2_O_5_ (180 mg, 1.0 mmol), imidazole (410 mg, 6.0 mmol), and H_2_O (500 mg, 278.0 mmol) in a mole ratio of 1:1:6:278 was sealed in a 23 ml Teflon-lined Parr reactor, which was placed in an oven and heated from room temperature to 393 K under autogenous pressure for 5 d. After cooling, the product was filtered off from the bright blue mother liquor, washed with distilled water and then dried overnight at room temperature. Dark-blue block-shaped crystals were easily separated from the residual uncharacterized brown-blue powder by hand under an optical microscope. The yield was 85% (0.15 g) based on V_2_O_5_. Analysis, calculated for C_12_H_16_CuN_8_O_6_V_2_: C 27.00, H 3.02, N 21.07%; found: C 27.01, H 3.08, N 21.02%.

### Refinement

H atoms were placed in geometrically idealized positions and constrained to ride on their parent atoms, with C—H = 0.95 and N—H = 0.88 Å and *U*_iso_(H) = 1.2*U*_eq_(C,N). In the final difference Fourier map, the maximum and minimum electron density of 0.51 and -0.30 eÅ^-3^ were located 0.77 and 0.33 Å from O2 and H45, respectively.

### Crystal data

**Table:**

[Cu_2_V_4_O_12_(C_3_H_4_N_2_)_8_]	*F*(000) = 1068
*M**_r_* = 1067.52	*D*_x_ = 1.806 Mg m^−^^3^
Monoclinic, *P*2_1_/*n*	Mo *K*α radiation, λ = 0.71073 Å
Hall symbol: -P 2yn	Cell parameters from 3583 reflections
*a* = 10.1761 (6) Å	θ = 2.4–27.4°
*b* = 16.5092 (9) Å	µ = 2.05 mm^−^^1^
*c* = 12.0372 (7) Å	*T* = 100 K
β = 103.844 (1)°	Block, dark blue
*V* = 1963.50 (19) Å^3^	0.24 × 0.20 × 0.18 mm
*Z* = 2	

### Data collection

**Table:**

Bruker APEX CCD diffractometer	4370 independent reflections
Radiation source: sealed tube	3830 reflections with *I* > 2σ(*I*)
Graphite monochromator	*R*_int_ = 0.025
ω and φ scans	θ_max_ = 28.2°, θ_min_ = 2.1°
Absorption correction: multi-scan (*SADABS*; Sheldrick, 1996)	*h* = −12→12
*T*_min_ = 0.639, *T*_max_ = 0.722	*k* = −20→16
11719 measured reflections	*l* = −15→15

### Refinement

**Table:**

Refinement on *F*^2^	Primary atom site location: structure-invariant direct methods
Least-squares matrix: full	Secondary atom site location: difference Fourier map
*R*[*F*^2^ > 2σ(*F*^2^)] = 0.028	Hydrogen site location: inferred from neighbouring sites
*wR*(*F*^2^) = 0.073	H-atom parameters constrained
*S* = 1.04	*w* = 1/[σ^2^(*F*_o_^2^) + (0.0408*P*)^2^ + 0.1105*P*] where *P* = (*F*_o_^2^ + 2*F*_c_^2^)/3
4370 reflections	(Δ/σ)_max_ = 0.001
262 parameters	Δρ_max_ = 0.51 e Å^−^^3^
0 restraints	Δρ_min_ = −0.30 e Å^−^^3^
0 constraints	

### Special details

**Table:**

Geometry. All e.s.d.'s (except the e.s.d. in the dihedral angle between two l.s. planes) are estimated using the full covariance matrix. The cell e.s.d.'s are taken into account individually in the estimation of e.s.d.'s in distances, angles and torsion angles; correlations between e.s.d.'s in cell parameters are only used when they are defined by crystal symmetry. An approximate (isotropic) treatment of cell e.s.d.'s is used for estimating e.s.d.'s involving l.s. planes.
Refinement. Refinement of *F*^2^ against all reflections. The weighted *R*-factor *wR* and goodness of fit *S* are based on *F*^2^, conventional *R*-factors *R* are based on *F*, with *F* set to zero for negative *F*^2^. The threshold expression of *F*^2^ > σ(*F*^2^) is used only for calculating *R*-factors(gt) *etc*. and is not relevant to the choice of reflections for refinement. *R*-factors based on *F*^2^ are statistically about twice as large as those based on *F*, and *R*- factors based on ALL data will be even larger.

### Fractional atomic coordinates and isotropic or equivalent isotropic displacement parameters (Å^2^)

**Table:**

	*x*	*y*	*z*	*U*_iso_*/*U*_eq_	
Cu1	0.80636 (2)	0.241362 (13)	0.801507 (19)	0.00947 (8)	
V1	0.61028 (3)	0.417383 (18)	0.91849 (3)	0.00910 (9)	
O1	0.64852 (14)	0.33438 (8)	0.85877 (11)	0.0151 (3)	
V2	0.46561 (3)	0.585997 (19)	0.82471 (3)	0.00889 (9)	
O2	0.75077 (14)	0.46664 (8)	0.97220 (12)	0.0183 (3)	
O3	0.50084 (14)	0.47895 (8)	0.80807 (11)	0.0143 (3)	
O4	0.47841 (14)	0.60729 (8)	0.97210 (11)	0.0151 (3)	
O5	0.31228 (14)	0.60726 (9)	0.74519 (12)	0.0162 (3)	
O6	0.92507 (14)	0.14149 (8)	0.71994 (12)	0.0158 (3)	
N11	0.82168 (16)	0.18449 (10)	0.95274 (13)	0.0131 (3)	
C12	0.9253 (2)	0.14465 (12)	1.01605 (17)	0.0151 (4)	
H12	1.0126	0.1424	1.0008	0.018*	
N13	0.89173 (18)	0.10785 (10)	1.10445 (14)	0.0167 (4)	
H13	0.9454	0.0780	1.1569	0.020*	
C14	0.7591 (2)	0.12508 (13)	1.09835 (18)	0.0204 (5)	
H14	0.7072	0.1075	1.1498	0.024*	
C15	0.7157 (2)	0.17227 (13)	1.00442 (17)	0.0199 (4)	
H15	0.6268	0.1935	0.9784	0.024*	
N21	0.78124 (16)	0.31036 (10)	0.66093 (13)	0.0123 (3)	
C22	0.77206 (19)	0.28959 (12)	0.55363 (16)	0.0145 (4)	
H22	0.7719	0.2353	0.5274	0.017*	
N23	0.76290 (17)	0.35500 (10)	0.48635 (14)	0.0167 (4)	
H23	0.7556	0.3550	0.4120	0.020*	
C24	0.7670 (2)	0.42209 (13)	0.55468 (18)	0.0186 (4)	
H24	0.7627	0.4772	0.5312	0.022*	
C25	0.7783 (2)	0.39402 (12)	0.66188 (18)	0.0169 (4)	
H25	0.7835	0.4267	0.7277	0.020*	
N31	0.97998 (16)	0.30136 (10)	0.86210 (13)	0.0116 (3)	
C32	1.0025 (2)	0.35656 (12)	0.94453 (16)	0.0132 (4)	
H32	0.9438	0.3674	0.9933	0.016*	
N33	1.11982 (16)	0.39489 (10)	0.94927 (14)	0.0140 (3)	
H33	1.1552	0.4333	0.9979	0.017*	
C34	1.1751 (2)	0.36370 (12)	0.86493 (17)	0.0146 (4)	
H34	1.2579	0.3793	0.8477	0.017*	
C35	1.08746 (19)	0.30603 (12)	0.81122 (16)	0.0139 (4)	
H35	1.0985	0.2740	0.7485	0.017*	
N41	0.64268 (16)	0.17312 (9)	0.73864 (13)	0.0113 (3)	
C42	0.51402 (19)	0.19205 (12)	0.72905 (16)	0.0135 (4)	
H42	0.4809	0.2457	0.7312	0.016*	
N43	0.43677 (17)	0.12534 (10)	0.71599 (14)	0.0151 (4)	
H43	0.3485	0.1239	0.7076	0.018*	
C44	0.5198 (2)	0.06006 (12)	0.71797 (17)	0.0166 (4)	
H44	0.4937	0.0047	0.7113	0.020*	
C45	0.6465 (2)	0.08987 (11)	0.73137 (17)	0.0136 (4)	
H45	0.7256	0.0584	0.7352	0.016*	

### Atomic displacement parameters (Å^2^)

**Table:**

	*U*^11^	*U*^22^	*U*^33^	*U*^12^	*U*^13^	*U*^23^
Cu1	0.01030 (13)	0.00903 (13)	0.00864 (13)	−0.00211 (8)	0.00144 (9)	0.00087 (8)
V1	0.01084 (17)	0.00739 (17)	0.00899 (16)	0.00014 (12)	0.00217 (12)	−0.00107 (11)
O1	0.0206 (7)	0.0109 (7)	0.0151 (7)	0.0012 (5)	0.0065 (6)	−0.0015 (5)
V2	0.00932 (16)	0.00903 (17)	0.00877 (16)	0.00116 (11)	0.00309 (12)	0.00181 (11)
O2	0.0156 (7)	0.0128 (7)	0.0247 (8)	−0.0018 (6)	0.0015 (6)	−0.0019 (6)
O3	0.0191 (7)	0.0112 (7)	0.0115 (7)	0.0019 (5)	0.0016 (5)	−0.0002 (5)
O4	0.0190 (7)	0.0147 (7)	0.0126 (7)	0.0012 (6)	0.0058 (6)	−0.0005 (5)
O5	0.0124 (7)	0.0221 (8)	0.0152 (7)	0.0040 (6)	0.0052 (6)	0.0041 (6)
O6	0.0156 (7)	0.0163 (7)	0.0176 (7)	−0.0010 (6)	0.0080 (6)	−0.0040 (6)
N11	0.0151 (8)	0.0135 (9)	0.0106 (8)	0.0002 (6)	0.0029 (7)	0.0005 (6)
C12	0.0160 (10)	0.0140 (10)	0.0133 (10)	−0.0005 (8)	−0.0002 (8)	0.0010 (7)
N13	0.0223 (9)	0.0130 (9)	0.0123 (8)	0.0001 (7)	−0.0011 (7)	0.0025 (6)
C14	0.0264 (12)	0.0220 (12)	0.0152 (10)	−0.0012 (9)	0.0101 (9)	0.0010 (8)
C15	0.0209 (11)	0.0247 (12)	0.0169 (10)	0.0034 (9)	0.0098 (9)	0.0041 (8)
N21	0.0137 (8)	0.0110 (8)	0.0120 (8)	−0.0019 (6)	0.0025 (6)	0.0004 (6)
C22	0.0144 (10)	0.0149 (10)	0.0139 (10)	−0.0012 (8)	0.0026 (8)	−0.0006 (8)
N23	0.0192 (9)	0.0206 (9)	0.0104 (8)	−0.0020 (7)	0.0038 (7)	0.0033 (7)
C24	0.0209 (11)	0.0140 (11)	0.0207 (11)	−0.0010 (8)	0.0048 (9)	0.0070 (8)
C25	0.0212 (11)	0.0102 (10)	0.0191 (10)	−0.0023 (8)	0.0041 (8)	0.0003 (8)
N31	0.0131 (8)	0.0116 (8)	0.0100 (8)	−0.0011 (6)	0.0024 (6)	0.0008 (6)
C32	0.0145 (10)	0.0128 (10)	0.0115 (9)	0.0000 (7)	0.0018 (8)	0.0008 (7)
N33	0.0158 (9)	0.0113 (8)	0.0135 (8)	−0.0030 (6)	0.0005 (7)	−0.0018 (6)
C34	0.0130 (10)	0.0152 (10)	0.0159 (10)	−0.0011 (8)	0.0044 (8)	0.0032 (8)
C35	0.0146 (10)	0.0165 (10)	0.0108 (9)	−0.0003 (8)	0.0031 (8)	−0.0007 (7)
N41	0.0125 (8)	0.0108 (8)	0.0105 (8)	−0.0009 (6)	0.0027 (6)	−0.0003 (6)
C42	0.0124 (9)	0.0143 (10)	0.0139 (10)	−0.0008 (8)	0.0032 (8)	−0.0012 (7)
N43	0.0109 (8)	0.0180 (9)	0.0169 (9)	−0.0020 (6)	0.0040 (7)	−0.0018 (7)
C44	0.0200 (10)	0.0117 (10)	0.0186 (10)	−0.0022 (8)	0.0056 (8)	−0.0003 (8)
C45	0.0167 (10)	0.0104 (9)	0.0142 (10)	0.0008 (7)	0.0047 (8)	−0.0001 (7)

### Geometric parameters (Å, º)

**Table:**

Cu1—N41	2.0033 (16)	N21—C25	1.381 (2)
Cu1—N31	2.0045 (16)	C22—N23	1.340 (3)
Cu1—N21	2.0049 (16)	C22—H22	0.9500
Cu1—N11	2.0209 (16)	N23—C24	1.374 (3)
Cu1—O6	2.3894 (13)	N23—H23	0.8800
Cu1—O1	2.4379 (13)	C24—C25	1.350 (3)
V1—O1	1.6370 (13)	C24—H24	0.9500
V1—O2	1.6373 (14)	C25—H25	0.9500
V1—O4^i^	1.8121 (13)	N31—C32	1.326 (2)
V1—O3	1.8253 (13)	N31—C35	1.378 (2)
V2—O6^ii^	1.6285 (14)	C32—N33	1.340 (2)
V2—O5	1.6611 (14)	C32—H32	0.9500
V2—O4	1.7826 (14)	N33—C34	1.373 (3)
V2—O3	1.8237 (14)	N33—H33	0.8800
O4—V1^i^	1.8122 (13)	C34—C35	1.358 (3)
O6—V2^iii^	1.6284 (14)	C34—H34	0.9500
N11—C12	1.319 (2)	C35—H35	0.9500
N11—C15	1.383 (3)	N41—C42	1.324 (2)
C12—N13	1.339 (3)	N41—C45	1.378 (2)
C12—H12	0.9500	C42—N43	1.340 (3)
N13—C14	1.364 (3)	C42—H42	0.9500
N13—H13	0.8800	N43—C44	1.366 (3)
C14—C15	1.357 (3)	N43—H43	0.8800
C14—H14	0.9500	C44—C45	1.354 (3)
C15—H15	0.9500	C44—H44	0.9500
N21—C22	1.318 (2)	C45—H45	0.9500
			
N41—Cu1—N31	174.98 (6)	C22—N21—C25	105.77 (16)
N41—Cu1—N21	94.25 (6)	C22—N21—Cu1	130.10 (14)
N31—Cu1—N21	87.11 (6)	C25—N21—Cu1	124.03 (13)
N41—Cu1—N11	87.53 (6)	N21—C22—N23	111.16 (18)
N31—Cu1—N11	91.75 (6)	N21—C22—H22	124.4
N21—Cu1—N11	172.34 (6)	N23—C22—H22	124.4
N41—Cu1—O6	85.01 (6)	C22—N23—C24	107.46 (17)
N31—Cu1—O6	90.13 (6)	C22—N23—H23	126.3
N21—Cu1—O6	91.15 (6)	C24—N23—H23	126.3
N11—Cu1—O6	96.43 (6)	C25—C24—N23	106.19 (18)
N41—Cu1—O1	85.21 (6)	C25—C24—H24	126.9
N31—Cu1—O1	99.72 (6)	N23—C24—H24	126.9
N21—Cu1—O1	85.48 (6)	C24—C25—N21	109.42 (18)
N11—Cu1—O1	87.25 (6)	C24—C25—H25	125.3
O6—Cu1—O1	169.40 (5)	N21—C25—H25	125.3
O1—V1—O2	108.20 (7)	C32—N31—C35	106.31 (16)
O1—V1—O4^i^	110.06 (7)	C32—N31—Cu1	126.24 (13)
O2—V1—O4^i^	111.36 (7)	C35—N31—Cu1	126.17 (13)
O1—V1—O3	108.31 (7)	N31—C32—N33	110.42 (17)
O2—V1—O3	109.51 (7)	N31—C32—H32	124.8
O4^i^—V1—O3	109.34 (6)	N33—C32—H32	124.8
V1—O1—Cu1	153.30 (8)	C32—N33—C34	108.05 (16)
O6^ii^—V2—O5	108.22 (7)	C32—N33—H33	126.0
O6^ii^—V2—O4	108.94 (7)	C34—N33—H33	126.0
O5—V2—O4	111.51 (7)	C35—C34—N33	106.05 (17)
O6^ii^—V2—O3	109.94 (7)	C35—C34—H34	127.0
O5—V2—O3	108.89 (7)	N33—C34—H34	127.0
O4—V2—O3	109.33 (6)	C34—C35—N31	109.17 (17)
V2—O3—V1	124.15 (7)	C34—C35—H35	125.4
V2—O4—V1^i^	138.46 (9)	N31—C35—H35	125.4
V2^iii^—O6—Cu1	167.49 (8)	C42—N41—C45	105.79 (16)
C12—N11—C15	105.61 (17)	C42—N41—Cu1	128.00 (13)
C12—N11—Cu1	129.10 (14)	C45—N41—Cu1	123.45 (13)
C15—N11—Cu1	124.94 (13)	N41—C42—N43	110.87 (17)
N11—C12—N13	111.40 (18)	N41—C42—H42	124.6
N11—C12—H12	124.3	N43—C42—H42	124.6
N13—C12—H12	124.3	C42—N43—C44	107.67 (17)
C12—N13—C14	107.45 (17)	C42—N43—H43	126.2
C12—N13—H13	126.3	C44—N43—H43	126.2
C14—N13—H13	126.3	C45—C44—N43	106.43 (18)
C15—C14—N13	106.57 (18)	C45—C44—H44	126.8
C15—C14—H14	126.7	N43—C44—H44	126.8
N13—C14—H14	126.7	C44—C45—N41	109.24 (17)
C14—C15—N11	108.97 (19)	C44—C45—H45	125.4
C14—C15—H15	125.5	N41—C45—H45	125.4
N11—C15—H15	125.5		
			
O2—V1—O1—Cu1	−1.75 (19)	N41—Cu1—N21—C25	127.77 (15)
O4^i^—V1—O1—Cu1	−123.65 (16)	N31—Cu1—N21—C25	−57.07 (15)
O3—V1—O1—Cu1	116.87 (16)	O6—Cu1—N21—C25	−147.14 (15)
N41—Cu1—O1—V1	179.21 (18)	O1—Cu1—N21—C25	42.92 (15)
N31—Cu1—O1—V1	0.15 (18)	C25—N21—C22—N23	−0.3 (2)
N21—Cu1—O1—V1	−86.13 (17)	Cu1—N21—C22—N23	−176.65 (13)
N11—Cu1—O1—V1	91.46 (17)	N21—C22—N23—C24	0.2 (2)
O6—Cu1—O1—V1	−157.9 (2)	C22—N23—C24—C25	−0.1 (2)
O6^ii^—V2—O3—V1	91.19 (10)	N23—C24—C25—N21	0.0 (2)
O5—V2—O3—V1	−150.41 (9)	C22—N21—C25—C24	0.2 (2)
O4—V2—O3—V1	−28.36 (11)	Cu1—N21—C25—C24	176.85 (14)
O1—V1—O3—V2	−164.38 (8)	N21—Cu1—N31—C32	106.98 (17)
O2—V1—O3—V2	−46.59 (11)	N11—Cu1—N31—C32	−65.43 (17)
O4^i^—V1—O3—V2	75.68 (10)	O6—Cu1—N31—C32	−161.87 (16)
O6^ii^—V2—O4—V1^i^	172.44 (11)	O1—Cu1—N31—C32	22.07 (17)
O5—V2—O4—V1^i^	53.06 (14)	N21—Cu1—N31—C35	−58.37 (16)
O3—V2—O4—V1^i^	−67.40 (13)	N11—Cu1—N31—C35	129.22 (16)
N41—Cu1—O6—V2^iii^	179.0 (4)	O6—Cu1—N31—C35	32.78 (16)
N31—Cu1—O6—V2^iii^	−2.2 (4)	O1—Cu1—N31—C35	−143.28 (15)
N21—Cu1—O6—V2^iii^	84.9 (4)	C35—N31—C32—N33	−1.0 (2)
N11—Cu1—O6—V2^iii^	−94.0 (4)	Cu1—N31—C32—N33	−168.67 (13)
O1—Cu1—O6—V2^iii^	156.1 (3)	N31—C32—N33—C34	0.8 (2)
N41—Cu1—N11—C12	124.78 (18)	C32—N33—C34—C35	−0.2 (2)
N31—Cu1—N11—C12	−50.25 (18)	N33—C34—C35—N31	−0.3 (2)
O6—Cu1—N11—C12	40.08 (18)	C32—N31—C35—C34	0.8 (2)
O1—Cu1—N11—C12	−149.90 (17)	Cu1—N31—C35—C34	168.52 (13)
N41—Cu1—N11—C15	−47.34 (17)	N21—Cu1—N41—C42	−76.98 (17)
N31—Cu1—N11—C15	137.63 (17)	N11—Cu1—N41—C42	95.55 (16)
O6—Cu1—N11—C15	−132.04 (16)	O6—Cu1—N41—C42	−167.77 (16)
O1—Cu1—N11—C15	37.98 (16)	O1—Cu1—N41—C42	8.11 (16)
C15—N11—C12—N13	0.1 (2)	N21—Cu1—N41—C45	124.56 (15)
Cu1—N11—C12—N13	−173.20 (13)	N11—Cu1—N41—C45	−62.91 (15)
N11—C12—N13—C14	−0.3 (2)	O6—Cu1—N41—C45	33.77 (14)
C12—N13—C14—C15	0.3 (2)	O1—Cu1—N41—C45	−150.35 (15)
N13—C14—C15—N11	−0.3 (2)	C45—N41—C42—N43	−0.1 (2)
C12—N11—C15—C14	0.1 (2)	Cu1—N41—C42—N43	−161.54 (13)
Cu1—N11—C15—C14	173.76 (14)	N41—C42—N43—C44	0.4 (2)
N41—Cu1—N21—C22	−56.42 (17)	C42—N43—C44—C45	−0.5 (2)
N31—Cu1—N21—C22	118.74 (17)	N43—C44—C45—N41	0.5 (2)
O6—Cu1—N21—C22	28.66 (17)	C42—N41—C45—C44	−0.3 (2)
O1—Cu1—N21—C22	−141.27 (17)	Cu1—N41—C45—C44	162.26 (13)

Symmetry codes: (i) −*x*+1, −*y*+1, −*z*+2; (ii) −*x*+3/2, *y*+1/2, −*z*+3/2; (iii) −*x*+3/2, *y*−1/2, −*z*+3/2.

### Hydrogen-bond geometry (Å, º)

Cg1, Cg2, Cg3 and Cg4 are the centroids of the C11/C12/N13/C14/C15, C21/C22/N23/C24/C25, C31/C32/N33/C34/C35 and C41/C42/N43/C44/C45 rings, respectively.

**Table:**

*D*—H···*A*	*D*—H	H···*A*	*D*···*A*	*D*—H···*A*
N13—H13···O3^iv^	0.88	2.01	2.827 (2)	155
N23—H23···O5^v^	0.88	1.95	2.779 (2)	155
N33—H33···O2^vi^	0.88	1.90	2.694 (2)	149
N43—H43···O5^vii^	0.88	1.88	2.701 (2)	155
C24—H24···*Cg*1^viii^	0.95	2.99	3.912 (2)	165
C12—H12···*Cg*2^iv^	0.95	2.59	3.429 (2)	147
C22—H22···*Cg*3^ix^	0.95	2.66	3.332 (2)	128
C44—H44···*Cg*3^iii^	0.95	2.91	3.816 (2)	161
C32—H32···*Cg*4^x^	0.95	2.76	3.321 (2)	119

Symmetry codes: (iii) −*x*+3/2, *y*−1/2, −*z*+3/2; (iv) *x*+1/2, −*y*+1/2, *z*+1/2; (v) −*x*+1, −*y*+1, −*z*+1; (vi) −*x*+2, −*y*+1, −*z*+2; (vii) −*x*+1/2, *y*−1/2, −*z*+3/2; (viii) −*x*−1/2, *y*+1/2, −*z*+3/2; (ix) *x*−1/2, −*y*+1/2, *z*−1/2; (x) *x*+1/2, −*y*−3/2, *z*+1/2.
